# Low on-treatment levels of serum soluble CD8 (sCD8) predict better outcomes in advanced non-small cell lung cancer patients treated with atezolizumab

**DOI:** 10.1007/s00262-023-03377-8

**Published:** 2023-01-23

**Authors:** Anna Siemiątkowska, Maciej Bryl, Katarzyna Kosicka-Noworzyń, Jakub Tvrdoň, Iwona Gołda-Gocka, Franciszek K. Główka

**Affiliations:** 1 Department of Physical Pharmacy and Pharmacokinetics, Poznan University of Medical Sciences, 3 Rokietnicka Street, 60-806, Poznań, Poland; 2 Department of Clinical Oncology with the Subdepartment of Diurnal Chemotherapy, Wielkopolska Center of Pulmonology and Thoracic Surgery, 62 Szamarzewskiego Street, 60-569 Poznań, Poland

**Keywords:** Immunotherapy, Biomarkers, Soluble proteins, PD-L1 inhibitor, Survival, NSCLC

## Abstract

**Background:**

Immunotherapy has changed the paradigm of treating non-small cell lung cancer (NSCLC). But, selecting patients who will achieve long-term benefits from treatment remains unsatisfactory. Here, we investigated the possible use of the soluble form of CD8 antigen (sCD8) in predicting durable disease control after PD-1/PD-L1 blockade. CD8 is a marker of the cytotoxic T lymphocytes. Its soluble form (sCD8) is secreted under activation of the immune system but also has immunosuppressive properties. The data about serum sCD8 in patients dosed with anti-PD-1/PD-L1 drugs are lacking.

**Methods and results:**

We included 42 NSCLC patients and collected samples at baseline and for the first 3 months of atezolizumab immunotherapy. The serum sCD8 concentrations were measured with the ELISA kit and correlated with treatment outcomes. Patients with durable (≥ 12 months) disease control presented lower serum sCD8 than those without long-term benefits. The sCD8 levels measured at the end of cycle 2 (sCD8.2) were the earliest time point that successfully differentiated patients (3.76 vs. 9.68 ng/mL, respectively, *p* < 0.001). Individuals with low sCD8.2 (≤ 4.09 ng/mL) presented longer progression-free survival (HR = 0.061, *p* < 0.001) and overall survival (HR = 0.104, *p* < 0.05) compared to individuals with high sCD8.2 (median values unreached vs. 4.4 months and 14.4 months for PFS and OS, respectively).

**Conclusions:**

Serum sCD8 could be an early biomarker of durable disease control after anti-PD-L1 treatment. Higher sCD8 in patients with worse outcomes could suggest the inhibitory effect of sCD8 on cytotoxic T-cells activation.

**Supplementary Information:**

The online version contains supplementary material available at 10.1007/s00262-023-03377-8.

## Introduction

Immunotherapy has changed the paradigm of treating non-small cell lung cancer (NSCLC). Introducing monoclonal antibodies targeting the programmed cell death 1 receptor/programmed cell death ligand-1 (PD-1/PD-L1) axis has significantly improved the treatment outcomes in both men and women [1]. However, the percentage of patients who do not respond to treatment is still high, with almost half of the patients presenting progression as their best response [2]. A recent meta-analysis showed that the median progression-free survival (PFS) was only 3.4 months, while the overall survival (OS)—10 months in real-world settings, with PD-1/PD-L1 inhibitors as the second-line therapy in advanced and metastatic NSCLC [2]. Thus, in the era of personalized medicine, the selection of patients who will achieve long-term benefits from anti-PD-1/PD-L1 therapy remains poor and unsatisfactory.

Over the last several years, multiple biomarkers have been tested to identify patients who would benefit from PD-1/PD-L1 inhibitors [3]. The only approved biomarker that has been included so far in the immunotherapy protocols, i.e., the expression of PD-L1 in lung cancer tissue, requires invasive biopsy. Moreover, the efficacy of this biomarker remains debatable, which implies that more specific and selective biomarkers are needed. Among others, the blood-based biomarkers are under intensive investigation as blood is easy to collect and enables multiple non-invasive sampling [4]. Here, we explored the usefulness of one another circulating molecule that has never been tested in relation to the PD-1/PD-L1 blockade, i.e., the soluble form of a cluster of differentiation 8 (CD8) protein (sCD8).

The CD8 antigen is a well-recognized marker of the cytotoxic T lymphocytes (CTLs, cytotoxic CD8^+^ T-cells) [5], which are considered a backbone of cancer immunotherapy [6]. The CD8 surface glycoprotein exists either as a heterodimer (built from one alpha and one beta chain) or a homodimer (composed of two alpha chains) and plays a crucial role in the neoplastic cells’ killing [5, 7]. Namely, a heterodimer CD8*αβ* acts as a co-receptor for the T-cell receptor (TCR): along with the TCR, it binds to the peptide-loaded major histocompatibility complex class I (MHC-I) at the surface of the antigen-presenting cells, which enhances the TCR signaling [5, 6] (see also Fig. 4a in the ‘Discussion’ section). The process initiates in the lymphoid tissue, where activated naïve CD8^+^ T-cells rapidly proliferate and differentiate into the effector CTLs, subsequently migrating into the target sites (e.g., the tumor microenvironment) [8, 9]. Conversely to CD8*αβ*, a homodimer CD8αα might negatively regulate the T-cell activation via being a TCR co-repressor [7]. Moreover, it modulates the activation of the natural killer cells through the inhibitory receptor KIR3DL1 [5].

Except for the membrane-bound CD8, also the soluble form of this protein has been identified, which originates from the alternative splicing [10] or membrane shedding [11]. Both the monomeric [10–12] and the dimeric forms [12–14] of sCD8 have been detected in human sera [12, 14] and in vitro experiments [10, 11, 13, 14]. The sCD8 is secreted upon the activation of CD8^+^ T lymphocytes [13, 15]; thus, its elevated levels might be a surrogate marker of immunological activation. In line with this hypothesis, some authors reported abnormal (too high) concentrations of sCD8 in conditions linked with the enhanced activity of the immune system, e.g., systemic lupus erythematosus [16, 17], rheumatoid arthritis [18], Graves’ disease [19], or acute renal allograft rejections [20]. On the other hand, the potential immunosuppressive role of sCD8 has been highlighted by others [21–24], suggesting its immunomodulatory character.

Even though immunotherapy aims to support the activation of the immune system [25], there are no published reports evaluating the role of sCD8 protein (i.e., a molecule clearly linked with the immune system) in the successful treatment with anti-PD-1/PD-L1 agents, neither in lung cancer nor in other malignancies. Thus, the focus of our study was to assess the usefulness of circulating sCD8 as a biomarker of long-term benefits from therapy with immune checkpoint inhibitors. We enrolled NSCLC patients treated with the anti-PD-L1 drug, atezolizumab (ATEZO), and prospectively collected several blood samples. The results of our study might serve as a basis for future research that would help to understand the exact role of serum sCD8 in the successful therapy with PD-1/PD-L1 inhibitors and—ultimately—improve treatment outcomes in cancer patients.

## Materials and methods

### Patient selection and study design

This was a prospective, observational, single-center study designed to evaluate the usefulness of selected serum soluble proteins as predictors of successful immunotherapy in patients with advanced NSCLC. Between February 2019 and June 2020, serial blood samples were collected at baseline and during the first 3 months of immunotherapy from 42 adult patients eligible for therapy with ATEZO within the Polish national NSCLC drug program. The detailed inclusion criteria for the program were described previously [26]. Subjects’ characteristic was presented in the ‘Results’ section. Patients received treatment at the Eugenia and Janusz Zeyland Wielkopolska Center of Pulmonology and Thoracic Surgery in Poznan, Poland. Participating in the project had no impact on medical decisions. The study was conducted in accordance with the Declaration of Helsinki and the approval of the Bioethics Committee at Poznan University of Medical Sciences (decisions 80/19 and 251/19). All subjects signed informed consent.

The anti-PD-L1 treatment (1200 mg of intravenous ATEZO every three weeks) was continued until disease progression, death, unacceptable (≥ grade 3) toxicity, or significant deterioration of the quality of life. Response to ATEZO was evaluated every 3 months according to the Response Evaluation Criteria in Solid Tumors (RECIST, v1.1) [27]. Clinical responses were categorized as complete response (CR), partial response (PR), stable disease (SD), or progressive disease (PD). Patients who continued therapy were followed up for at least 24 months from the start of treatment. The primary endpoint was the **long-term disease control rate**, i.e., the percentage of patients with at least SD after 12 months from the start of treatment (patients with SD, PR, or CR who were still on ATEZO treatment at the evaluation time point, i.e., one year from the start of immunotherapy). The secondary endpoints were: **progression-free survival** (PFS), defined as the time from the first ATEZO dose to confirmed progression or death, and **overall survival** (OS), defined as the time from the start of immunotherapy to death.

### Sample collection

Blood samples (*n* = 173) were collected at baseline (first day of ATEZO administration, pre-dose) and at the end of cycles 1–4. After clotting, serum was separated by centrifugation at 1700 × g for 15 min, aliquoted, and kept at − 80 °C until used.

### Determination of serum sCD8 levels

The concentrations of sCD8 were determined by sandwich ELISA using a kit from MyBioSource (San Diego, CA, USA; Cat. No. MBS016364). All experiments were performed according to the manufacturer’s instructions. The optical density was read at 450 nm with the BioTek 800TS plate reader (BioTek Instruments, USA), and the log–log regression was used to calculate the sCD8 levels. The calibration range was 3.12–100 ng/mL. Samples were prepared in duplicate, and their mean was used for statistical analyses.

### Statistical analysis

The statistical analysis was performed with MedCalc 20.106 software (MedCalc Software Ltd, Ostend, Belgium). For all analyses, a *p* value < 0.05 was considered significant. The normal distribution of variables was assessed with the Shapiro–Wilk test; the normally distributed data were then compared with the t-Student test and expressed as mean ± standard deviation (sd), while the non-normally distributed data with Mann–Whitney U test and expressed as median (interquartile range). Categorical data were compared with Fisher’s exact test and expressed as numbers (%). The changes in sCD8 level over time compared to baseline were assessed with the Wilcoxon test.

The receiver operator characteristic curve (ROC) analysis with the Youden index was used to determine the optimal cutoff for the sCD8 protein. The durable disease control, i.e., at least SD 12 months from the start of ATEZO treatment, was chosen as an outcome for ROC analysis. The Kaplan–Meier method was then implemented to investigate the association between low/high sCD8 levels and progression or survival, and the differences between the groups were assessed with a log-rank test. Subjects who did not experience the event of interest (death or progression for analyses with OS and PFS, respectively) were censored in Kaplan–Meier analyses. The exception was patients who died within the next 3 months after the last dose of ATEZO—all these patients were considered progressed regardless of the reason for ending immunotherapy. The univariable and multivariable Cox proportional hazard models were used to confirm the prognostic value of sCD8 protein on OS and PFS. All variables from the univariable Cox analyses with a *p* < 0.1 were included in the multivariable model.

## Results

### Baseline patients’ characteristics

**Table ****1** presents the characteristics of the study population. The baseline data were retrospectively extracted from the hospital records and a short questionnaire completed by participants on the day of enrollment. All individuals were of Caucasian origin and had undergone one prior systemic treatment. The vast majority of patients were diagnosed with adenocarcinoma (64.3%) and stage IV of the disease (95.2%). Following the requirements of the NSCLC drug program in Poland, patients with adenocarcinoma did not have mutations in *EGFR* (epidermal growth factor receptor) or *ALK* (anaplastic lymphoma kinase) genes. The PD-L1 status was unknown for most participants as PD-L1 expression in cancer tissue does not play a role in the qualification for ATEZO treatment (supplementary file in ref. [26]).

**Table 1:** Baseline characteristics of the study population

Age [years]	65.1 ± 6.3
Age ≥ 65	23 (54.8%)
BMI [kg/m^2^]^#^	26.6 ± 4.3
BMI ≥ 25	23 (56.1%)
Males	27 (64.3%)
NSCLC subtype	
Adenocarcinoma	27 (64.3%)
Squamous cell carcinoma	11 (26.2%)
Other	4 (9.5%)
Smoking status^&^	
Never smokers^a^	4 (10.3%)
Former smokers^b^	28 (71.8%)
Current smokers^c^	7 (17.9%)

Data are presented as mean ± sd or number of patients (%); data from ^#^1 and ^&^3 patients were missing

^a–c^Subjects with a lifetime smoking of ^a^ < 100 cigarettes or ^b,c^ ≥ 100 cigarettes who have ^b^quit or ^c^continued smoking at the time of enrollment

### Clinical benefits and survival

**Fig. 1:**
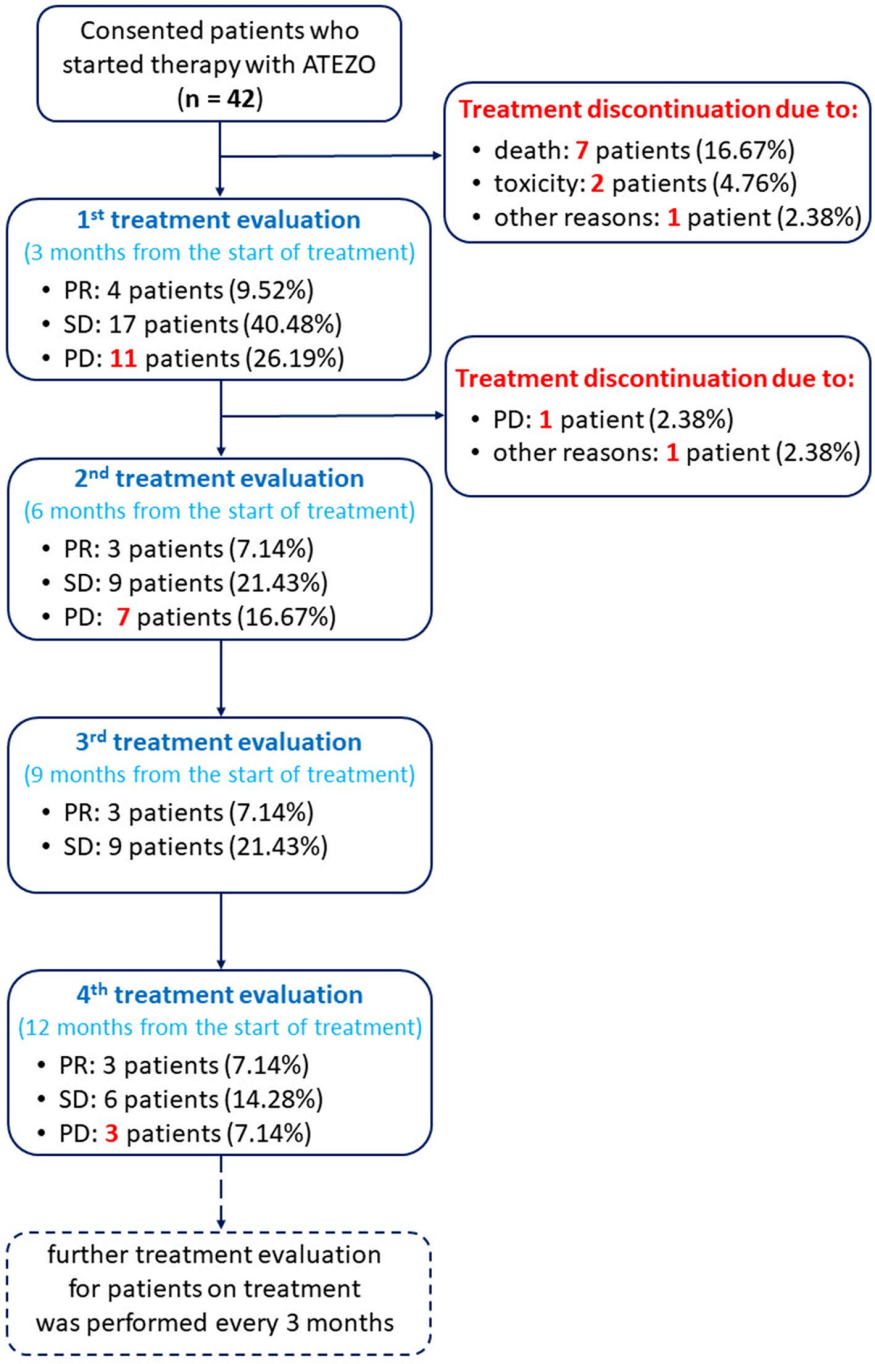
Flowchart of response to ATEZO in 42 NSCLC patients included in the study. Patients who stopped immunotherapy are highlighted in red. *ATEZO* atezolizumab, *NSCLC* non-small cell lung cancer, *PD* progressive disease, *PR* partial response, *SD* stable disease

Figure 1 demonstrates the results of the treatment response recorded during the study period. Short-term clinical benefits (evaluated 3 months from the start of treatment) were observed in 50.0% of patients, while durable disease control (≥ 12 months)—in 21.4%. Almost 40% of patients who showed benefits (at least SD) at their first response evaluation progressed within the next 3 months. The objective response rate was 14.3%, but PR lasted only 3 months in one patient, while in the other two, it was delayed ≥ 12 months. Five patients (11.9%) continued ATEZO immunotherapy for more than two years.

Two patients (4.8%) stopped immunotherapy due to severe (grade ≥ 3) toxicity (both received only one dose of ATEZO), and the other two (4.8%) due to progression and toxicity. Twenty-two patients (52.4%) discontinued treatment due to confirmed progression, three (7.1%)—because of the unacceptable quality of life, and eight (19.0%)—due to death, including seven individuals who died before their first response evaluation. The median PFS in the whole study population was 4.5 (3.0–12.1) months, and the median OS was 16.8 (4.6–28.7) months. At the time of data analysis, five patients (11.9%) were still on the ATEZO treatment, and twelve patients (28.6%) were alive. One subject (2.4%) was lost to follow-up after discontinuation of ATEZO immunotherapy.

### Determination of sCD8 levels

In total, 173 serum samples were analyzed for sCD8 concentrations. The sCD8 levels in two samples (1.2%) exceeded the upper limit of quantitation (100 ng/mL); thus, a new (previously unfrozen) aliquot of those sera was diluted per the manufacturer’s protocol and re-analyzed. Thirteen samples (7.5%) were below the lower limit of quantitation (3.12 ng/mL), with the range of the observed concentrations 2.16–3.08 ng/mL and a median value of 2.59 ng/mL (95% confidence interval, 95% CI: 2.44–2.85 ng/mL). The sCD8 concentration in all BQL (below the quantification limit) samples was at least twice the kit’s sensitivity (1 ng/mL); therefore, we decided to include all BQL samples in statistical tests. As discussed in literature [28], BQL data carry relevant information, so ignoring those samples, especially in the biomarker study, could bias the obtained results.

We decided to exclude four samples from statistical analysis; the reason was a long delay in the next dose of ATEZO in one patient, which could have affected the sCD8 concentrations. The second dose of ATEZO was administered to this patient ten instead of three weeks after the first dose; thus, only the baseline serum sample from this patient (sCD8.0) was used in statistics.

### Serum sCD8 level in the study population

The median baseline sCD8 level (sCD8.0) was 7.44 (4.45–14.58) ng/mL in all patients. After the start of immunotherapy, the median sCD8 concentrations were: 9.51 (4.62–14.67) ng/mL at the end of cycle one (sCD8.1); 6.66 (4.64–14.25) ng/mL at the end of cycle two (sCD8.2); 6.00 (4.14–16.70) ng/mL at the end of cycle three (sCD8.3), and 7.41 (5.21–14.16) ng/mL at the end of cycle four (sCD8.4), i.e., about 3 months from the start of treatment.

The sCD8 concentrations at baseline and during immunotherapy were independent of age (< 65 vs. ≥ 65), BMI (< 25 vs. ≥ 25), gender, NSCLC subtype (adenocarcinoma vs. others), and smoking status (current smokers vs. others) (Supplementary Materials, Fig. S1–S5).

### Serum sCD8 levels and long-term disease control

**Fig. 2:**
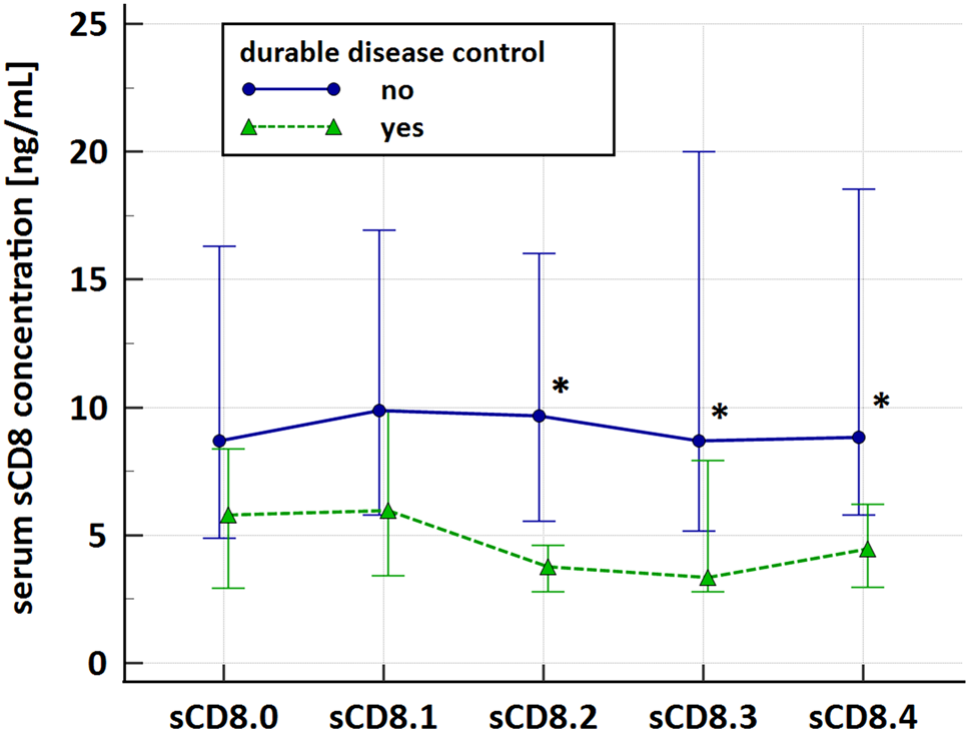
Serum sCD8 during the first 3 months of immunotherapy with atezolizumab (1200 mg Q3W) in relation to long-term (≥ 12 months) disease control. Data are presented as medians (interquartile ranges). Patients with long-term benefits from immunotherapy are marked with triangles/dashed lines, while those who lacked long-term benefits from treatment are marked with circles/solid lines. Time points with a significant difference between the groups are highlighted with asterisks. Specific time points referred to: sCD8.0—baseline, sCD8.1 to sCD8.4—concentrations of sCD8 at the end of cycles 1–4; disease control was defined as at least stable disease

Figure 2 presents the changes in the sCD8 levels within the first 3 months of ATEZO treatment in relation to durable (≥ 12 months) disease control. Baseline sCD8 values were not different between the groups. After the start of immunotherapy, there were no significant changes in the sCD8 concentrations compared to baseline in either group (*p* > 0.05 from the Wilcoxon test). However, patients who achieved long-term disease control presented significantly lower sCD8 concentrations at the end of cycles 2 (sCD8.2), 3 (sCD8.3), and 4 (sCD8.4) than patients who lacked the long-term benefits from treatment (*p* < 0.05 from Mann–Whitney U test). The median sCD8 values were: 5.81 versus 8.68 ng/mL at baseline (*p* > 0.05); 5.97 versus 9.89 ng/mL (*p* = *0.06*) for sCD8.1; 3.76 versus 9.68 ng/mL (*p* < 0.001) for sCD8.2; 3.36 versus 8.70 ng/mL (*p* < 0.05) for sCD8.3; 4.48 versus 8.85 ng/mL (*p* < 0.05) for sCD8.4. There were no differences in sCD8 levels between patients who achieved and lacked the objective response during immunotherapy (data not shown).

### Cutoff selection

As sCD8 concentrations at the end of the second cycle were the earliest time point that successfully identified patients with durable disease control, the sCD8.2 levels were selected for survival analysis. The ROC analysis was performed to find the best sCD8.2 value to predict the long-term benefits from treatment (supplementary Fig. S6). We found sCD8.2 level ≤ 4.09 ng/mL (AUC = 0.928, 95% CI: 0.785–0.988, *p* < 0.001) as a threshold with the highest sensitivity (75.0%, 95% CI: 34.9–96.8%) and specificity (96.2%, 95% CI: 80.4–99.9%), and further classified patients into low- and high-sCD8.2 level subgroups (*n* = 7 and *n* = 27, respectively). The Kaplan–Meier and Cox proportional hazard regression analyses were subsequently performed to test the usefulness of the ROC-derived cutoff for PFS and OS.

### Survival analysis

**Fig. 3:**
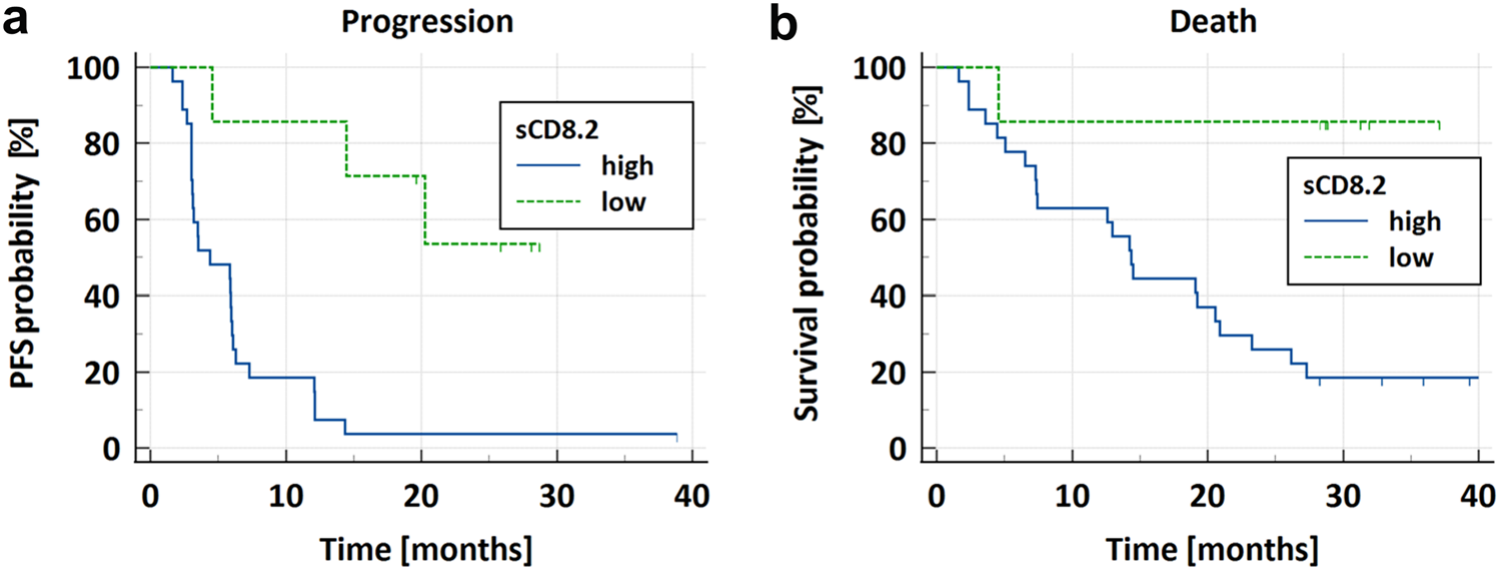
Kaplan–Meier curves for **a** progression-free survival (PFS) and **b** overall survival (OS) in non-small cell lung cancer patients dosed with atezolizumab stratified by high (> 4.09 ng/mL) and low (≤ 4.09 ng/mL) serum sCD8 levels at the end of cycle 2 (sCD8.2). Log-rank test showed significant differences in PFS (*p* < 0.001) and OS (*p* = 0.007) between patients with low- and high-sCD8.2

Kaplan–Meier analyses confirmed better outcomes in subjects with low sCD8.2 concentrations (Fig. 3). Patients with high sCD8.2 (> 4.09 ng/mL) achieved a median PFS of 4.4 months (95% CI: 3.0–6.0 months) and a median OS of 14.4 months (95% CI: 7.3–20.9 months), while patients with low sCD8.2 (≤ 4.09 ng/mL) reached neither the median PFS nor OS (with a 95% CI follow-up for this subgroup 16.3–34.5 months).

The univariable Cox proportional hazard analyses (Table 2) confirmed the low sCD8.2 level as a prognostic factor of longer PFS and OS. For PFS, the multivariable Cox regression was also performed to account for the other covariates’ influence. Patients with low sCD8 concentrations at the end of cycle 2 presented 93.9% lower hazard of progression (adjusted HR = 0.061, 95% CI: 0.012–0.304, *p* < 0.001), and 89.6% lower hazard of death (HR = 0.104, 95% CI: 0.014–0.777, *p* < 0.05) compared to patients with high sCD8.2.

**Table 2:** The univariable Cox regression analyses of progression-free survival and overall survival in non-small cell lung cancer patients treated with atezolizumab

	Progression-free survival		Overall survival	
	HR [95% CI]	*p* value	HR [95% CI]	*p* value
Age ≥ 65	0.692 [0.333–1.440]	0.325	0.725 [0.319–1.646]	0.442
BMI ≥ 25	0.961 [0.451–2.045]	0.917	0.752 [0.325–1.741]	0.506
Female gender	0.944 [0.443–2.009]	0.881	0.845 [0.357–1.997]	0.701
Adenocarcinoma	0.810 [0.372–1.761]	0.595	0.563 [0.242–1.313]	0.183
Current smoker	**2.932 [1.078–7.979]**	**0.035**	1.653 [0.558–4.893]	0.364
Objective response	**0.320 [0.096–1.068]**	***0.064***	0.618 [0.183–2.085]	0.438
Low sCD8.2 (≤ 4.09 ng/mL)	**0.140 [0.039–0.495]**	**0.002**	**0.104 [0.014–0.777]**	**0.027**

Bolded results have a *p* value of < 0.1

*BMI* body mass index, *CI* confidence interval, *HR* hazard ratio, *sCD8.2* serum sCD8 levels at the end of cycle 2

## Discussion

This is the first study to demonstrate the circulating soluble sCD8 protein as a predictor of successful anti-PD-1/PD-L1 therapy in cancer. NSCLC patients with durable (≥ 12 months) disease control presented lower on-treatment levels of serum sCD8 compared to those who lacked the long-term benefits from treatment. We identified the sCD8 concentrations measured at the end of cycle 2 to be predictive in terms of treatment efficacy: individuals with low sCD8.2 (≤ 4.09 ng/mL) presented longer PFS (adjusted HR = 0.061, *p* < 0.001) and OS (HR = 0.104, *p* < 0.05) compared to individuals with high sCD8.2. Thus, sCD8 seems to be a promising early biomarker of successful NSCLC therapy with ATEZO. Further studies are warranted to confirm our observation in a larger cohort of patients, in other types of cancer, and other PD-1/PD-L1 inhibitors.

Data regarding sCD8 protein and lung cancer are scarce. The serum sCD8 levels remain unchanged in NSCLC compared to healthy controls [29–31] and do not depend on the disease stage (III or IV) [29] or NSCLC subtype [29, 30]. Our study confirmed this observation: we found no differences in sCD8 levels between patients with adenocarcinoma and other NSCLC subtypes (supplementary Fig. S4). Moreover, the protein levels were similar in females and males (supplementary Fig. S3), normal weight versus overweight/obese individuals (supplementary Fig. S2), and younger and older patients (supplementary Fig. S1), which also corroborates with the available literature [14, 32, 33]. Active smoking did not impact the sCD8 levels (supplementary Fig. S5), which supports the observations from the general population [34] and contradicts those presented for pregnant women [35].

We demonstrated that NSCLC patients who achieved long-term disease control presented lower serum sCD8 at the end of cycles 2–4. Although no published papers assessed the circulating sCD8 levels in cancer patients on PD-1/PD-L1 inhibitors, lower baseline sCD8 was previously shown in responders to immunotherapy with interferon-alpha (IFN-α) [36] and higher on-treatment sCD8—in patients with progressive disease dosed with IFN-γ [37]. In contrast, other authors highlighted better outcomes with a higher baseline sCD8 [38] or a higher increase in serum sCD8 during treatment [39], but those patients were dosed with a different drug (interleukin-2, IL-2). The ambiguous results could suggest that the direction of correlation between sCD8 levels and immunotherapy success is not that obvious and might depend on additional factors, such as a type of malignancy and the used immunomodulator. Accordingly, higher sCD8 levels were favorable in melanoma and renal cell carcinoma patients but only during treatment with IL-2 [38, 39] and not with IFN-γ [37]. Moreover, different behavior of sCD8 was noted during immunotherapy with various drugs: there was a trend toward the sCD8 levels increasing after dosing with recombinant IL-2 [31, 39–41] or successful treatment with IFN-γ [37], but a similar trend was not observed in our cohort (Fig. 2). Interestingly, in patients on IFN-α, the sCD8 concentrations increased or decreased after successful immunotherapy depending on the initial sCD8 values [36].

Poor anti-PD-L1 treatment outcomes in patients with high serum sCD8 might be due to the inhibitory effect of sCD8 on immunological activation. Indeed, despite considering the soluble form of CD8 as a marker of immunological activation (e.g., [13, 15, 17, 20]), sCD8 also inhibited (in a dose-dependent manner [42]) the CTLs in vitro [22, 42] and attenuated the function of CD8^+^ T-cells in vivo [21]. A potential immunosuppressive role of sCD8 was previously suggested in some autoimmunological diseases, e.g., rheumatoid arthritis [18] or Hashimoto’s disease [24, 43]. In rheumatoid arthritis, despite the elevated sCD8 levels (reflecting, most likely, the immunological activation), the increase in sCD8 preceded the clinical improvement (suggesting the suppression of the immunological response) [18]. Similarly, patients with severe Hashimoto’s disease presented lower serum sCD8 compared to the mild form of the disease [24], and those with thyrotoxicosis (i.e., a condition in which thyrocytes are getting damaged mostly by cytotoxic T-cells) showed lower sCD8 compared to healthy controls [43].

**Fig. 4:**
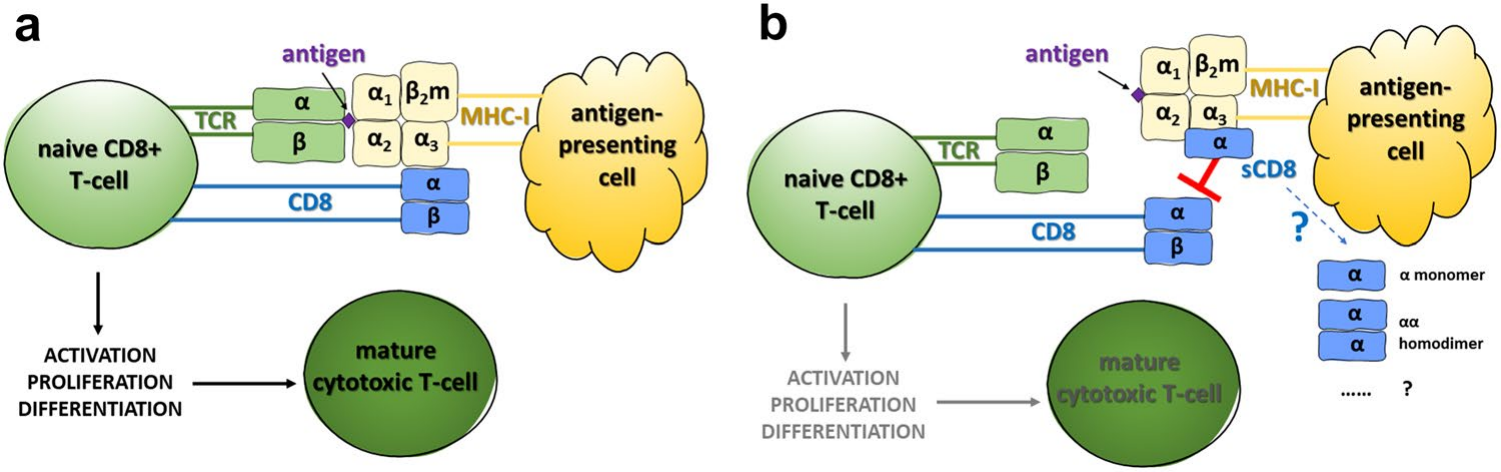
Schematic presentation of the interaction between TCR and MHC-I during antigen presentation **a** in case of a proper (i.e., undisturbed) activation of the immune system **b** proposed mechanism in case of the sCD8 excess. **a** MHC-I (built from a three-domain *α* chain and a *β*_2_m chain) presents a peptide antigen to a heterodimeric TCR; the CD8 heterodimer serves as a co-receptor for TCR: it enhances T-cell antigen recognition by binding to MHC-I [6]. **b** The sCD8 protein binds to MHC-I, which might block the interaction between membrane-bound CD8 and MHC-I; as various sCD8 forms have been reported by different authors in human blood (for further details, please go to the main text), at the moment it is unclear which specific sCD8 form interacts with MHC-I. *β*_2_*m* *β*_2_-microglobulin, *CD8* cluster of differentiation 8, *MHC-I* major histocompatibility complex class I, *sCD8* soluble CD8 protein, *TCR* T-cell receptor

The CD8 antigen serves as a co-receptor for TCR (Fig. 4a): it enhances the binding of MHC-I to TCR and increases TCR signaling [5], so the efficient (undisturbed) interaction between membrane-bound CD8 and MHC-I seems to be necessary to properly activate the naïve CD8^+^ T-cells. On the other hand, the sCD8 protein binds to MHC-I [42] and HLA-I (i.e., a ‘human version’ of MHC) [23], which may interfere with the binding of the CTL-derived membrane-bound CD8 to MHC-I/HLA-I (Fig. 4b). This interaction, in turn, could result in an ineffective antigen presentation and failed immunological activation [23], which may be the case in our patients who lacked the long-term benefits from treatment. As immunotherapy works through the activation of the immune system (and, compared to chemotherapy, does not kill cancer cells directly), any disturbances in the function of immune system might lead to worse treatment outcomes in patients dosed with PD-1/PD-L1 inhibitors.

The interesting question remains which sCD8 form is mainly secreted in NSCLC patients undergoing immunotherapy with PD-1/PD-L1 inhibitors. The in vitro studies showed that the molecular weight of sCD8 secreted by the human T-cell lines was 27 kDa [11], 30 kDa (a form generated via the alternative splicing) [10], 52 kDa [14], or 54 kDa [13], indicating both the monomeric and dimeric forms. Moreover, Pui and colleagues [14] demonstrated the elevated levels of 52 kDa homodimer of sCD8 in lymphoid malignancies, while Schlesinger and colleagues [12] revealed that three different sCD8 forms were detected in human sera, i.e., 28–30 kDa, 57–62 kDa, and 66–70 kDa, the last one more widely distributed in the HIV-positive individuals. This observation suggests that a different proportion of various sCD8 molecules might be secreted under specific clinical conditions, e.g., in patients with the chronically stimulated immune system. The ELISA kit used by our research team had both the capture and detection antibody directed against CD8α, suggesting that it could—hypothetically—detect sCD8α monomer as well as sCD8 multimers, depending on the binding site.

### Strengths and limitations

We acknowledge that our study had some limitations. Firstly, due to its pilot character, the sample size was small, and we could not include a validation cohort. Moreover, we did not define an adequately powered sample size a priori as the study was a pioneer in demonstrating the usefulness of sCD8 as a biomarker of successful anti-PD-L1 therapy. Also, the number of samples collected from specific patients was sometimes incomplete, as a few patients died or stopped receiving ATEZO before their first response evaluation. Lastly, the biological mechanism underlying better outcomes in patients with low sCD8 levels has not been investigated, and further experiments are necessary to reveal the role of sCD8 in successful cancer immunotherapy. If poor immunotherapy outcomes resulted from the inhibitory effect of sCD8 during T-cells activation, the agents selectively directed against sCD8 could potentially reverse that effect. This question, at the moment, remains open.

Despite all these limitations, our pilot study demonstrated, for the first time, that circulating sCD8 levels could successfully indicate patients with durable disease control after PD-L1 blockade and that the topic is worth exploring. The strength of the study was its longitudinal character, which allowed us to identify the earliest time point that differentiated patients into those with and without long-term benefits based on the sCD8 levels. Also, the vast majority of samples were collected before the COVID-19 pandemic outbreak, while the sample collection process was definitely ended before the number of COVID-19 cases in Poland became significant; thus, our results were unaffected by SARS-CoV-2 infection. As sCD8 increases in viral diseases (elevated levels were observed, e.g., in mononucleosis [44] and dengue hemorrhagic fever [45]), this condition was mandatory to obtain the unbiased results.

## Conclusions

The serum on-treatment levels of sCD8 could be considered an early biomarker of durable (≥ 12 months) disease control in patients treated with anti-PD-L1 drugs. Low sCD8 after two cycles of ATEZO predicted longer PFS and OS in NSCLC, suggesting a possible inhibitory effect of high sCD8 concentrations on the activation of the immune system. Further studies are warranted to confirm our observations in a larger study population, as well as in patients receiving other anti-PD-1/PD-L1 agents and suffering from other types of cancer. However, the results of our study are promising and provide a step toward a better understanding of phenomena associated with successful anti-PD-1/PD-L1 immunotherapy.

### Supplementary Information

Below is the link to the electronic supplementary material.

Supplementary file1 (PDF 591 KB)

## Data Availability

All relevant data are included in this article and presented as figures, tables, or supplementary materials. The data are available from the corresponding author upon reasonable request.

## Abbreviations

*ALK*: Anaplastic lymphoma kinase gene
ATEZO: Atezolizumab
BMI: Body mass index
BQL: Below the quantification limit
CD8: Cluster of differentiation 8
CI: Confidence interval
CR: Complete response
CTLs: Cytotoxic T lymphocytes
*EGFR*: Epidermal growth factor receptor gene
HIV: Human immunodeficiency virus
HLA-I: Human leukocyte antigen class I
HR: Hazard ratio
IFN: Interferon
IL-2: Interleukin-2
MHC-I: Major histocompatibility complex class I
NSCLC: Non-small cell lung cancer
OS: Overall survival
PD: Progressive disease
PD-1: Programmed cell death 1 receptor
PD-L1: Programmed cell death ligand-1
PFS: Progression-free survival
PR: Partial response
RECIST: Response Evaluation Criteria in Solid Tumors
ROC: Receiver operator characteristic curve
sCD8: Soluble form of CD8 antigen
SD: Stable disease
sd: Standard deviation
TCR: T-cell receptor
