# Supplementary material for: Low on-treatment levels of serum soluble CD8 (sCD8) predict better outcomes in advanced non-small cell lung cancer patients treated with atezolizumab
Source: Cancer Immunol Immunother. 2023 Jan 23;72(6):1853–63. doi: 10.1007/s00262-023-03377-8 (PMC9870198; doi:10.1007/s00262-023-03377-8)
Supplement: Supplementary file 1 — Supplementary file1 (PDF 591 KB) [file 262_2023_3377_MOESM1_ESM.pdf]

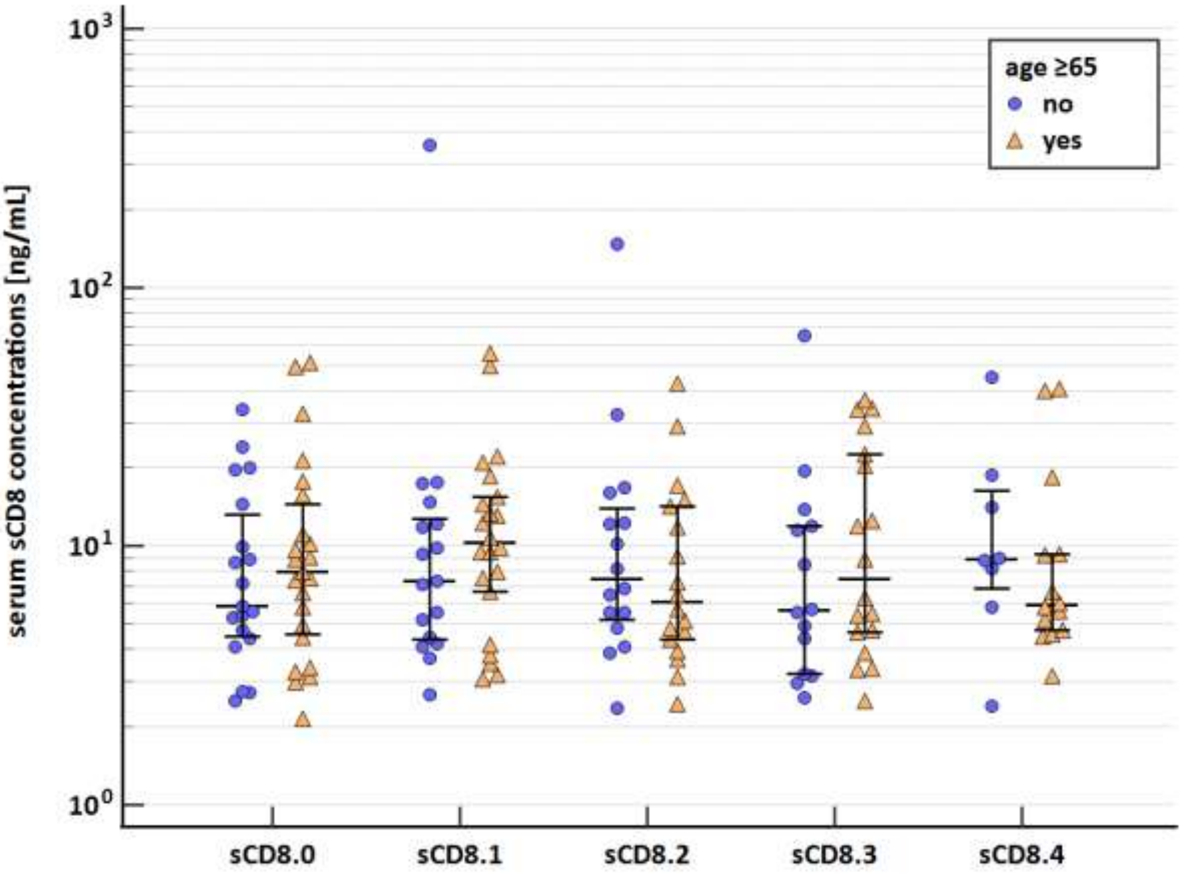

Fig. S1. Serum soluble CD8 (sCD8) levels stratified by age.

Figure presents the observed sCD8 concentrations (circles and triangles) along with their medians (horizontal lines) and interquartile ranges (whiskers) at baseline (sCD8.0) and at the end of cycles 1 (sCD8.1), 2 (sCD8.2), 3 (sCD8.3), and 4 (sCD8.4) in younger (<65 years) and older (≥65 years) non-small cell lung cancer patients treated with atezolizumab (1200 mg Q3W). Results are presented on the logarithmic scale due to the wide range of the observed values; however, raw (untransformed) data were used in statistical analyses. Mann-Whitney U test showed no significant differences in the observed sCD8 concentrations between younger and older patients at any of the tested time points ($p>0.05$).

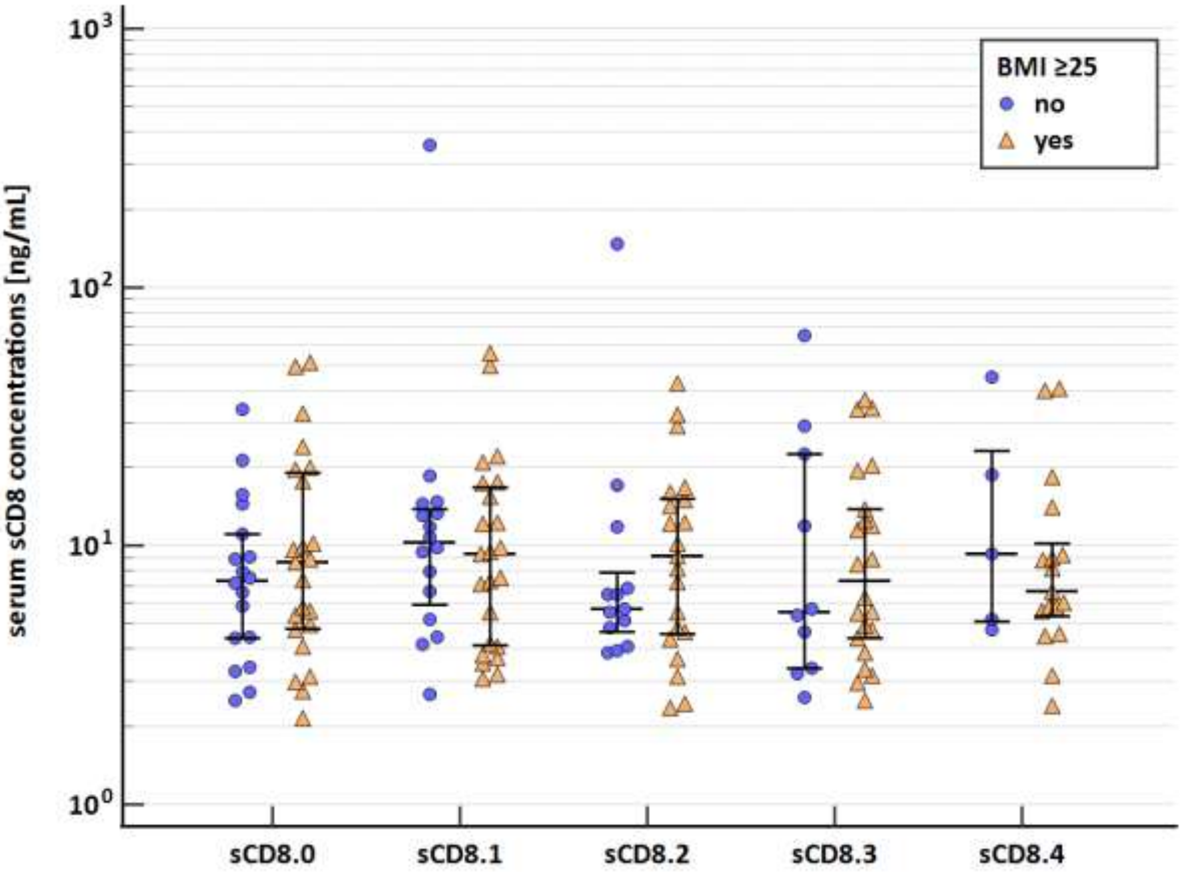

Fig. S2. Serum soluble CD8 (sCD8) levels stratified by BMI.

Figure presents the observed sCD8 concentrations (circles and triangles) along with their medians (horizontal lines) and interquartile ranges (whiskers) at baseline (sCD8.0) and at the end of cycles 1 (sCD8.1), 2 (sCD8.2), 3 (sCD8.3), and 4 (sCD8.4) in normal weight (BMI <25) and overweight/obese (BMI ≥25) non-small cell lung cancer patients treated with atezolizumab (1200 mg Q3W). None of the patients in the study population was underweight (BMI <18.5). Results are presented on the logarithmic scale due to the wide range of the observed values; however, raw (untransformed) data were used in statistical analyses. Mann-Whitney U test showed no significant differences in the observed sCD8 concentrations between normal weight and overweight/obese patients at any of the tested time points ($p>0.05$).

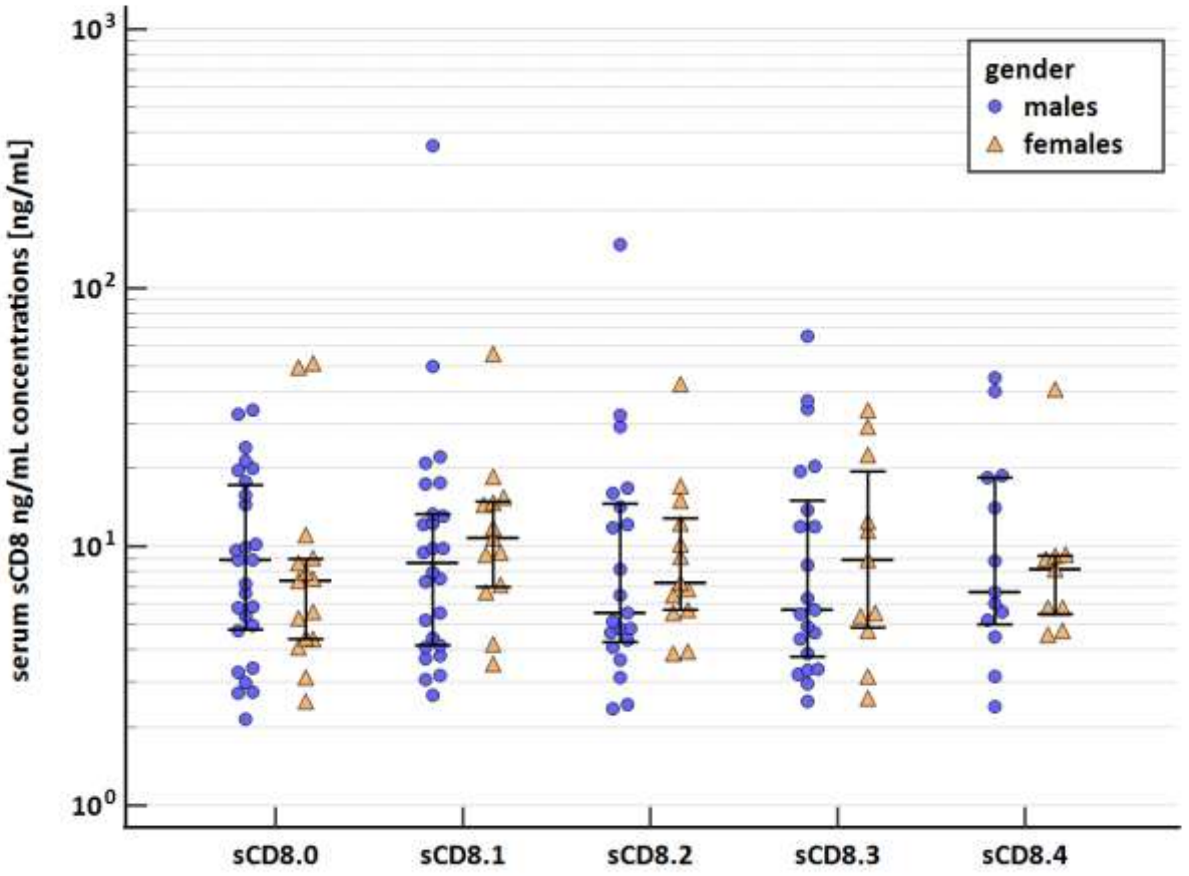

Fig. S3. Serum soluble CD8 (sCD8) levels stratified by gender.

Figure presents the observed sCD8 concentrations (circles and triangles) along with their medians (horizontal lines) and interquartile ranges (whiskers) at baseline (sCD8.0) and at the end of cycles 1 (sCD8.1), 2 (sCD8.2), 3 (sCD8.3), and 4 (sCD8.4) in female and male non-small cell lung cancer patients treated with atezolizumab (1200 mg Q3W). Results are presented on the logarithmic scale due to the wide range of the observed values; however, raw (untransformed) data were used in statistical analyses. Mann-Whitney U test showed no significant differences in the observed sCD8 concentrations between females and males at any of the tested time points ($p>0.05$).

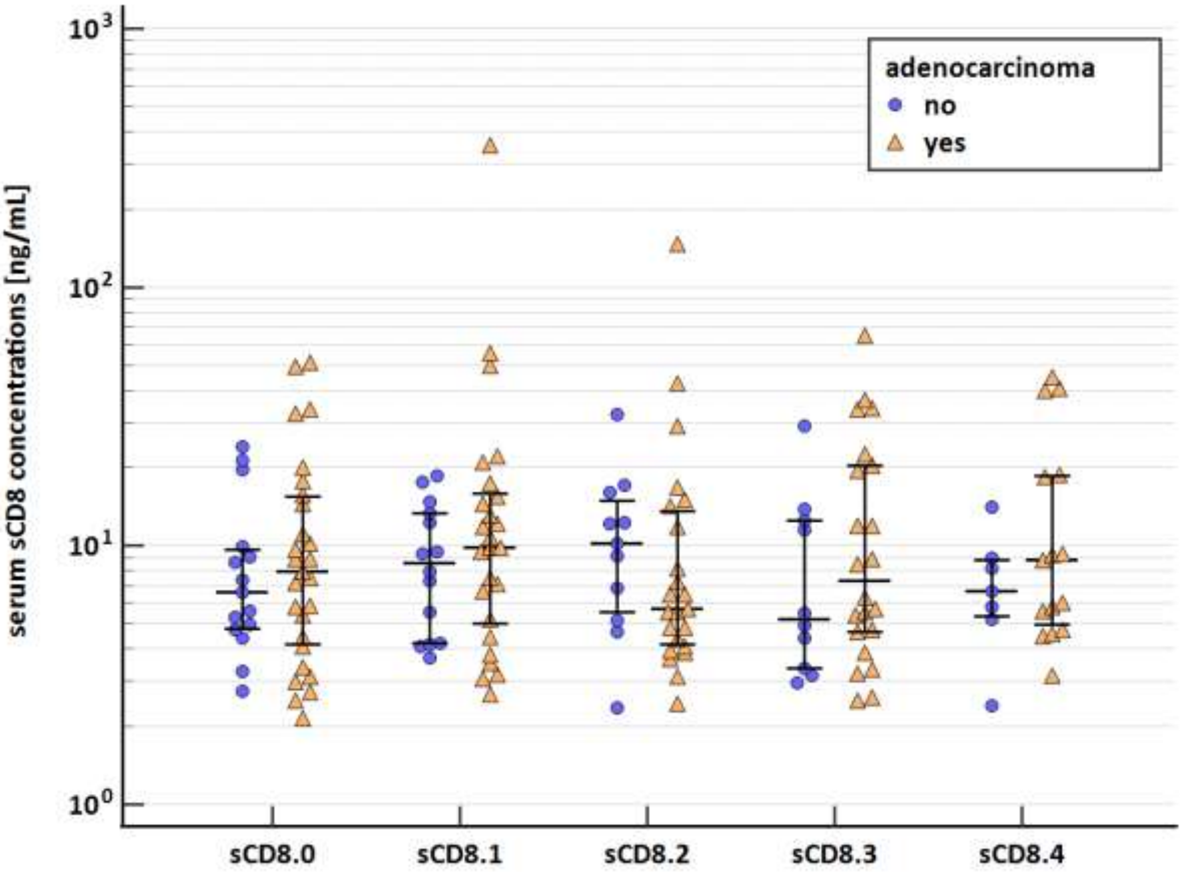

Fig. S4. Serum soluble CD8 (sCD8) levels stratified by NSCLC subtype.

Figure presents the observed sCD8 concentrations (circles and triangles) along with their medians (horizontal lines) and interquartile ranges (whiskers) at baseline (sCD8.0) and at the end of cycles 1 (sCD8.1), 2 (sCD8.2), 3 (sCD8.3), and 4 (sCD8.4) in adenocarcinoma and non-adenocarcinoma non-small cell lung cancer patients treated with atezolizumab (1200 mg Q3W). Results are presented on the logarithmic scale due to the wide range of the observed values; however, raw (untransformed) data were used in statistical analyses. Mann-Whitney U test showed no significant differences in the observed sCD8 concentrations between patients with adenocarcinoma and other NSCLC subtypes at any of the tested time points ($p>0.05$).

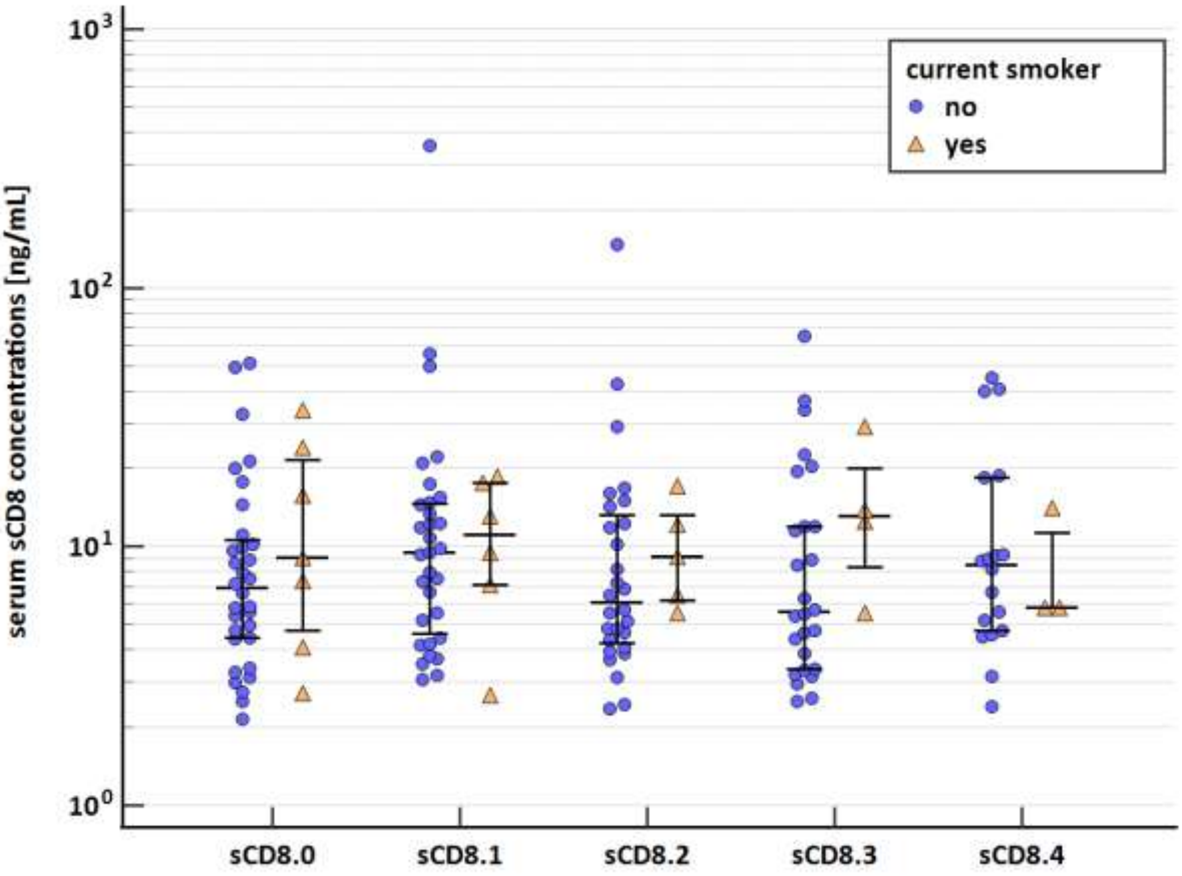

Fig. S5. Serum soluble CD8 (sCD8) levels stratified smoking status.

Figure presents the observed sCD8 concentrations (circles and triangles) along with their medians (horizontal lines) and interquartile ranges (whiskers) at baseline (sCD8.0) and at the end of cycles 1 (sCD8.1), 2 (sCD8.2), 3 (sCD8.3), and 4 (sCD8.4) in non-small cell lung cancer patients treated with atezolizumab (1200 mg Q3W) who smoked and did not smoke at the time of enrollment. Results are presented on the logarithmic scale due to the wide range of the observed values; however, raw (untransformed) data were used in statistical analyses. Mann-Whitney U test showed no significant differences in the observed sCD8 concentrations between current smokers and other patients at any of the tested time points ($p>0.05$).

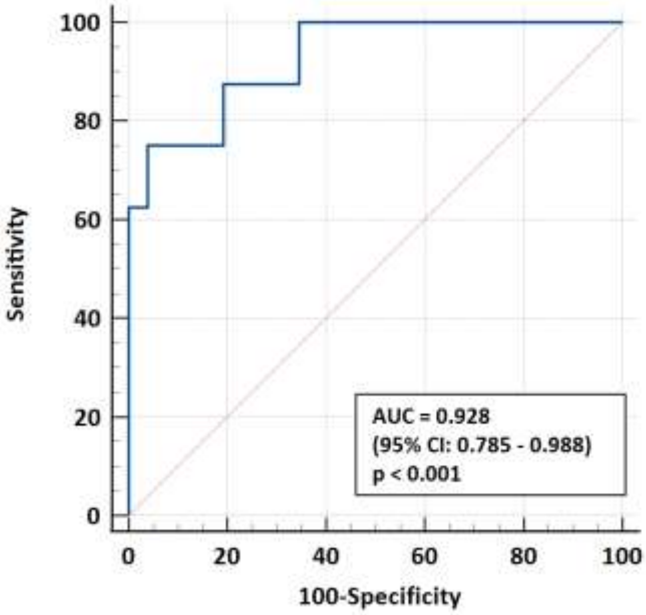

Fig. S6. The ROC curve for serum sCD8 at the end of cycle 2 (sCD8.2) and durable (≥12 months) disease control in NSCLC patients treated with atezolizumab.

In accordance with the Youden index (0.712), sCD8.2 concentration ≤4.09 ng/mL was found as the best cut-off point with sensitivity 75.0% (95% CI: 34.9 – 96.8%) and specificity 96.2% (95% CI: 80.4 – 99.9%).

Abbreviations: AUC, area under the curve; 95% CI, 95% confidence interval; NSCLC, non-small cell lung cancer; ROC, receiver operating characteristic; sCD8, soluble CD8. Durable disease control was defined as at least stable disease confirmed by imagining tests.
